# Spontaneous Resolution of Ventricular Pre-Excitation During Childhood: A Retrospective Study

**DOI:** 10.3390/jcm14072367

**Published:** 2025-03-29

**Authors:** Antonio Sanzo, Alessandro Seganti, Andrea Demarchi, Riccardo Simone Fino, Irene Raso, Alessia Claudia Codazzi, Barbara Petracci, Andrea Bongiorno, Roberto Rordorf, Savina Mannarino

**Affiliations:** 1 Arrhythmia and Electrophysiology Unit, Division of Cardiology, Fondazione IRCCS Policlinico S. Matteo, 27100 Pavia, Italy; antonio.sanzo@gmail.com (A.S.);; 2 Department of Molecular Medicine, University of Pavia, 27100 Pavia, Italy; 3 Cardiocentro Ticino Institute, Ente Ospedaliero Cantonale, 6900 Lugano, 6500 Bellinzona, Switzerland; andrea.demarchi@eoc.ch; 4 Independent Researcher, 20100 Milano, Italy; riccardo.s.fino@gmail.com; 5 Cardiology Unit, Paediatric Department, Buzzi Children’s Hospital Milano, 20154 Milano, Italy; irene.raso@asst-fbf-sacco.it (I.R.);; 6 Paediatric Cardiology Department, Fondazione IRCCS Policlinico San Matteo, 27100 Pavia, Italy

**Keywords:** ventricular pre-excitation, pediatric electrophysiology, sudden cardiac death, arrhythmic risk stratification, Wolf–Parkinson–White syndrome, arrhythmias

## Abstract

**Background/Objectives:** Ventricular pre-excitation (VP) increases the risk of sudden cardiac death among children. While transcatheter ablation could potentially be therapeutic, it is not without risk, especially in smaller children. Accessory pathways (APs) may spontaneously lose anterograde conduction properties over time, making invasive treatment unnecessary. We aim to investigate the probability of spontaneous loss of VP during childhood, as well as the potential factors that may be associated with VP resolution. **Methods:** We conducted a retrospective study of patients with VP diagnosed before 12 years of age and referred to two Northern Italian tertiary care hospitals between 1993 and 2021. Patients with complex congenital heart disease were excluded. Our primary objective was to determine the likelihood of spontaneous resolution of VP. **Results:** Overall, 153 patients were included, with a median age at first diagnosis of 4.9 years (25th–75th percentile: 75 days–8.4 years) and a median follow-up of 4.9 years (25th–75th percentile: 1.8–8 years). Through left truncated Kaplan–Meier analysis, we estimated that anterograde conduction would persist in 53% and 33.8% of patients at the age of 1 and 16 years, respectively. Our findings revealed that the absence of symptoms and intermittent VP were associated with a higher likelihood of VP resolution. It is noteworthy that no major arrhythmic events were reported. **Conclusions:** Our study strongly supports the implementation of a conservative strategy in younger children with VP. Our findings indicate that a significant proportion of pediatric patients may experience spontaneous resolution of VP in the early years of their lives, making any invasive treatment unnecessary.

## 1. Introduction

Ventricular pre-excitation (VP) is a congenital condition characterized by the conduction of impulses from the atrial to the ventricular myocardium through an accessory pathway (AP) in addition to conduction across the atrioventricular (AV) node. The prevalence of this condition is estimated to be between 0.1% and 0.3% [1,2]. For a long time, it has been studied in the context of Wolf–Parkinson–White (WPW) syndrome, which is characterized by VP and arrhythmia-related symptoms. VP has been associated with a higher risk of sudden cardiac death (SCD) of about 0.15% per year and may approach 3–4% over a lifetime, particularly during childhood and adolescence [3]. Transcatheter ablation of the AP is the cornerstone treatment as it has been shown to be highly effective with low risk in adults [4]. However, transcatheter ablation may carry a higher risk of complications with slightly lower efficacy in the first years of life, particularly in low-weight children (less than 15 kg) [5,6]. As a result, recent European guidelines indicate that transcatheter ablation should only be considered after the age of 8 [4].

The hypothesis that patients younger than 21 years carry a higher risk of major arrhythmic events (MAEs) is supported by previous studies that also included patients with congenital heart disease (CHD—a subgroup with an inherently higher risk profile) [7,8]. Moreover, some of those patients were treated with outdated pharmacological therapies such as blockers of the AV node, which ultimately favors anterograde conduction over the AP, and only a minority of patients under the age of 8 were included. It is known that during growth, a proportion of APs may spontaneously lose anterograde conduction, particularly before the first year of age. However, the natural history of VP in childhood remains poorly understood and, given the conflicting literature, determining the most suitable treatment for these patients remains a challenge [9,10,11]. Our study aims to describe the likelihood of the persistence of anterograde conduction from birth to the end of the pediatric period in a contemporary pediatric cohort without CHD, analyzing the factors that may influence the spontaneous resolution of VP. The primary endpoint of the study was the spontaneous loss of VP, defined as the disappearance of the manifest delta wave on at least two consecutive electrocardiograms (ECGs) and/or on one 24 h Holter monitoring ECG. We also describe the management strategy we pursued in these patients.

## 2. Methods

As part of this study, we retrospectively recruited patients diagnosed with VP between ages 0 and 12 years and referred to two high-volume, tertiary care hospitals in Lombardy, Italy (Fondazione Policlinico San Matteo, Pavia, and Ospedale dei Bambini “Vittore Buzzi”, Milano), from 1993 to 2021. The review board of both hospitals approved the publication of the anonymized retrospective data of the patients, using information collected for routine clinical practice, and waived the requirement for specific informed consent; however, since these are research hospitals, each parent or guardian signed a generic consent form for their child to participate in clinical research using anonymized data. The study was conducted in accordance with the Declaration of Helsinki Patient age at diagnosis was defined by the earliest available pre-excited electrocardiogram. The patients enrolled were evaluated and the diagnostic ECG was performed as part of screening for long QT syndrome, screening for sports medicine, preoperative screening, symptoms, or as a recommendation by pediatricians. Patients were enrolled through an electrocardiogram performed either for the presence of symptoms or as part of ECG screening.

All ECGs were reviewed by at least two investigators to confirm the diagnosis of VP and estimate the location of the APs following the Arruda algorithm [12]. All patients underwent an echocardiogram to exclude CHD. The duration of follow-up was defined as the time from presentation to the last clinical follow-up. Spontaneous loss of VP was defined as the absence of the delta wave on a 24 h Holter ECG, ensuring a reliable assessment of conduction loss through the accessory pathway (AP). Patients with spontaneous loss of VP underwent annual re-evaluation and if a delta wave reappeared on subsequent assessments, they were reassigned to the persistent VP group.

Follow-up ended when patients reached the age of 16 or were lost to follow-up.

Patients with significant or complex CHD and double APs, either suspected during non-invasive screening (ECG and 24 h Holter ECG) or confirmed during an invasive electrophysiological study, were excluded.

Data collected included demographics, medical history, presence of persistent or intermittent VP, spontaneous resolution of manifest VP, and clinical events during follow-up. Atrial fibrillation (AF) with rapid conduction over an AP, arrhythmic syncope, SCD, and death were considered major arrhythmic events (MAEs).

### 2.1. Risk Stratification Tools

Risk stratification for arrhythmia was performed using both non-invasive and invasive methods. Non-invasive risk stratification was conducted using resting a 12-lead ECG, 24 h Holter ECG, and exercise stress testing. All patients underwent 24 h Holter ECG both at baseline and during follow-up, while the exercise stress test was only performed on subjects deemed able to cooperate in the performance of the test. Individuals who showed both pre-excited and non-pre-excited beats on the 24 h Holter ECG were considered to have intermittent VP. Patients with baseline intermittent VP on Holter ECG or abrupt beat-to-beat loss of VP during the exercise test were considered to possess non-invasive, low-risk characteristics. In the present paper, these patients are indicated as “low risk” to simplify readability.

Invasive risk stratification was performed using either transesophageal or intracavitary electrophysiological studies. Individuals with accessory pathway refractoriness at baseline greater than 250 ms or a shortest pre-excited RR interval (SPERRI) greater than 250 ms, with refractoriness during beta-adrenergic stimulation greater than 220 ms or a SPERRI greater than 220 ms, were considered not at high risk [13,14], even though it is known that non-invasive stratification tools in pediatric patients do not demonstrate very high negative predictive value [15,16].

### 2.2. Management

Patients were managed following the most recent Consensus Statements and Clinical practice guidelines [6,13,17]. Chronic drug therapy was initiated in symptomatic patients when needed. Transcatheter ablation was performed when the risk–benefit ratio was considered acceptable as stated by international documents and after acceptance of informed consent provided by the parents or a legal guardian.

### 2.3. Statistical Analysis

Given the time-to-event dependency of the congenital condition under study and because the observation often started after birth, we considered that the left truncation resulted from the design of the study to delineate the survival curve of VP [18,19]. The results are presented as the mean ± SD for normally distributed continuous variables (tested with the Shapiro–Wilk normality test), as the median (25th and 75th percentiles) for continuous variables without normal distribution, and as numbers (%) for categorical data. One-way analysis of variance and Student’s unpaired *t*-test were used to compare normally distributed continuous variables. For non-normally distributed continuous variables, the Wilcoxon rank-sum test was used. Chi-square and Fisher’s exact tests were used to compare categorical variables. By the term “event”, we considered the occurrence of the spontaneous loss of VP, and by the term “survival”, we meant the persistence of manifest VP. Patients that underwent effective catheter ablation were right-truncated. Freedom from events was reported using the Kaplan–Meier method and comparisons were performed using the log-rank test. A two-tailed *p*-value < 0.05 was considered statistically significant. Univariate analysis of predictors of the primary endpoint was performed with Cox proportional hazards regression. All variables with a *p*-value < 0.10 and those considered clinically relevant were inserted into a multivariate Cox regression model to assess the hazard ratio (HR) and 95% confidence intervals (CIs) of the relationship between predictors and the primary endpoint. The rule of limiting the number of independent variables to 1 for every 10 events was followed. All statistical analyses, including Kaplan–Meier curves and the graphs, were performed with the use of R Project software (version 4.2.2) and its package “ggsurvfit” and “gtsummary”.

## 3. Results

### 3.1. Patient Characteristics

In this study, 153 patients were enrolled with a median age of 4.9 years (range: 75 days–8.4 years) at the first diagnosis; of these patients, 64 (41.9%) were female. The median follow-up time was 4.9 years (range: 1.6–8 years). Table 1 summarizes the main population characteristics. According to electrocardiographic criteria [12], the most common location of the AP insertion was the right free wall and the right posteroseptal area (26.6% each), as detailed in Table 1.

Arrhythmia-related symptoms were reported in 30.7% of children or by their caregivers. The most common symptom was palpitations, occurring in 32 cases (68%), followed by acute heart failure during SVT in 7 cases (14.9%). Notably, patients who experienced acute heart failure during SVT were predominantly younger children, particularly infants. Other reported symptoms included vomiting in three cases (6.3%), and chest pain, dizziness, poor oral intake, or slow growth in one case each (2.1%). None of the patients experienced syncope, chronic heart failure, or symptoms related to AF. Tachyarrhythmias were documented in 31 patients (20.3%), all of which were orthodromic atrioventricular re-entrant tachycardia (AVRT). Neither antidromic AVRT nor atrial fibrillation (AF) was observed in our study. No cases of antidromic tachycardia were detected.

None of the included patients had significant CHD. There were 10 (6.5%) patients with non-complex CHD, specifically small self-resolving ventricular septal defects, and hemodynamically insignificant atrial septal defects. In eight patients (5.2%), echocardiographic evaluation revealed interventricular dyssynchrony, which was attributed to accessory pathway conduction. These children were monitored with serial echocardiograms, and none developed chronic ventricular dysfunction. At the time of the electrophysiological study, the ejection fraction values were within the normal range (>55%), despite the presence of echocardiographic evidence of ventricular dyssynchrony. Interventricular dyssynchrony was associated with the following locations of the AP: anteroseptal (four patients), right free wall (three patients), and right posteroseptal (one patient). Among patients with interventricular dyssychrony, the accessory pathway disappeared spontaneously in two cases at the age of 266 and 3028 days, respectively, and ablation was performed in three patients at the age of 10.3, 11.7, and 12.5 years, respectively.

### 3.2. Resolution of Ventricular Pre-Excitation

**Figure 1:**
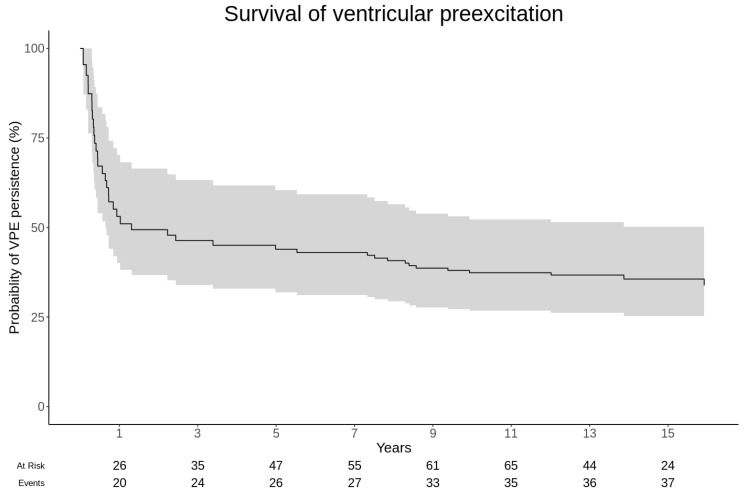
The left-truncated survival analysis curve of manifest pre-excitation.

During follow-up, spontaneous resolution of VP was observed in 42 (28%) cases. The left-truncated survival analysis curve of manifest VP is depicted in Figure 1. It estimates that the probability of persistence of anterograde conduction is 53.1% (40.0–70.2) at one year of life, 40.7% (29.4–56.5) at 8 years, and 33.8% (23.7–48.4) at 16 years.

**Figure 2:**
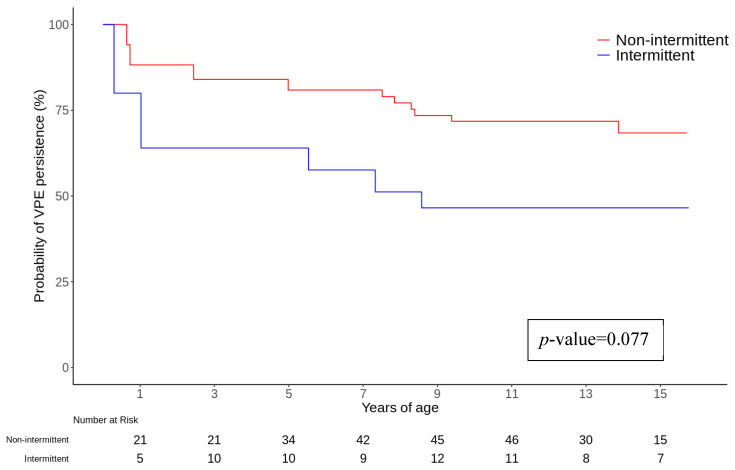
Kaplan–Meier survival curves for VP persistence.

Sex, AP localization, “low risk” characteristics at non-invasive risk stratification tests, ECG documentation of SVT, and the necessity to start chronic pharmacological therapy were not found to be associated with the primary endpoint in univariate and multivariate analyses and Cox regression analysis. However, the evidence of intermittent VP reached statistical significance during multivariate analysis and was associated with a higher probability of spontaneous loss of VP (HR 3.45, CI 1.32–9.05, *p*-value 0.012) (Figure 2). Furthermore, the presence of symptoms was related to a higher risk of persistence of VP during follow-up during both univariate (HR 0.43, CI 0.20–0.93, *p*-value 0.032) and multivariate analyses (HR 0.15, CI 0.03–0.68, *p*-value 0.014), as described in Table 2 and Figures S1–S3. Notably, palpitations were reported by 76% of patients with persistent VP and 25% of those with spontaneous loss of VP (*p* < 0.001), regardless of ECG evidence of SVT.

At the end of the follow-up, none of the children experienced any major arrhythmic event, and all were alive at the end of the study.

### 3.3. Risk Stratification

Non-invasive risk stratification was performed in 112 (73.2%) patients, and an invasive stratification study was performed in 46 (30%) patients. In total, 67 (43.8%) children presented low-risk characteristics during the non-invasive risk stratification tests. Among them, 1 (1.5%) patient experienced SVT during the follow-up period and 16 (23.9%) patients received treatment. Among patients deemed to be at high risk, five (11.1%) experienced SVT, and four (8.9%) received chronic treatment.

Additionally, 46 patients (30%) underwent an electrophysiological study, with 9 patients undergoing a transesophageal electrophysiological study (EPS) and 42 undergoing an intracardiac EPS. Unfortunately, electrophysiological data were available for only 20 patients, among whom 11 had high-risk characteristics, making this dataset uninformative regarding the overall outcome. The selection between transesophageal and intracavitary EPS was primarily influenced by patient age and cooperation, as transesophageal EPS was preferred for younger or less cooperative children to minimize the invasiveness of the procedure. In contrast, intracavitary EPS was directly performed in patients at high arrhythmic risk to allow for a more comprehensive evaluation of accessory pathway conduction properties and, when indicated, facilitate ablation within the same procedure.

### 3.4. Chronic Drug Therapy

Twenty-seven symptomatic children required chronic antiarrhythmic therapy. The first therapeutic strategy was adequate in controlling the symptoms in 74% of cases. If the first therapy was ineffective, a second (22.3%) and possibly a third strategy (3.7%) were attempted. Flecainide was the most prescribed drug, alone or in combination (75% of treated patients), at a median age of 6.1 years. Flecainide was the first-line treatment in most cases, while beta-blockers, primarily propranolol, were preferred for younger children. In more complex cases, amiodarone and sotalol were considered as second- or third-line options. In cases where the first treatment failed to achieve adequate symptom control, additional therapeutic combinations were explored, including beta-blockers such as propranolol or atenolol, amiodarone in selected cases with careful monitoring for potential side effects, and sotalol for patients with recurrent, high-risk supraventricular tachycardia (SVT). Overall, approximately 26% of patients required a change in therapy after the initial treatment due to insufficient symptom control. A complete list of the treatments is summarized in Table 3.

### 3.5. Catheter Ablation

Catheter ablation was performed in 30 (19.6%) subjects and effective in 23/30 (76.6%), with the earliest ablation performed at 6.6 years, apart from an urgent procedure attempted in a 2-year-old child with acute heart failure due to drug-refractory SVT. The median time from diagnosis to ablation was 5.1 years (2.7–6.1). The indications were symptoms for 6 patients, high-risk characteristics at invasive stratification in 14, SVT in 6, and the presence of interventricular dyssynchrony in 3.

## 4. Discussion

The best management strategy for VP in childhood remains unresolved. Therefore, understanding the probability of spontaneous loss of anterograde conduction along the AP is of the utmost importance in decision-making for this cohort of patients. Moreover, little is known about the natural history of VP in this period of life. Our study showed that the probability of spontaneous loss of VP is high in the first year of life (47%). Even though the odds are lower in the subsequent years, they are not null (up to 66% at 16 years of age in the entire population). In addition, the absence of symptoms and an intermittent VP were associated with a higher likelihood of spontaneous loss of VP, even after correction for age.

An important consideration is that not all accessory pathways behave similarly, particularly in their ability to regress over time. It has been hypothesized that APs responsible for SVT possess stronger antegrade conduction properties and shorter refractory periods, making them less likely to regress spontaneously. This may explain why symptomatic patients, irrespective of ECG evidence of SVT, in our study had a lower probability of VP resolution, reinforcing the notion that AP conduction properties influence its natural history. These findings align with prior observations suggesting that APs with higher conduction capacities and lower refractory periods are more resistant to spontaneous resolution.

Our use of the left-truncated Kaplan–Meier method offers a nuanced view that accounts for the time-dependent nature of the condition, enhancing our understanding of the natural resolution rates of VP across different ages. It suggests a more continuous assessment of risk, which is crucial for developing long-term management strategies in pediatric patients diagnosed with WPW syndrome. These results significantly add new insights for the management of children with VP detected in very early childhood. Differing from previous studies, we enrolled consecutive patients with a mean age of 5 years without severe CHD, referred to two tertiary care hospitals in the last 30 years and treated following the most recent evidence. We report the youngest median age ever presented in the literature: Santinelli et al. enrolled patients aged between 5 and 8 years old (median age 10 years) [9]; Cain et al. [20] enrolled around 30% of pts under 1 year of age, but the average age at diagnosis was 7 years. The remaining studies primarily involved an adult population. Another peculiarity is that the intentional exclusion of complex CHD helped to outline the survival profile of VP in the normal heart. These population characteristics, along with the relatively small sample size, may explain the absence of AF, syncope, and SCD. The rarity of SCD and the safety of treatment has already been described in several previous small studies [17].

Our results align closely with subgroup analysis in Cain et al.’s and Benson et al.’s studies, where patients enrolled at an age younger than three months experienced a disappearance of VP in around 35% of patients with a shorter follow-up. In other studies, the percentage of spontaneous resolution was found to be lower as a consequence of enrolment at school age or later and due to a shorter follow-up.

Perry et al. examined the phenomenon of early disappearance and late recurrence of WPW syndrome in children, highlighting that supraventricular tachycardia associated with WPW syndrome that begins in the first year of life may disappear in 93% of cases; however, there is a risk of recurrence later in childhood, typically after 8 years of age. This study involved a significant proportion of patients with CHD (37%), 23% of whom had Ebstein’s anomaly, underscoring the varied clinical trajectories in this population. These findings align with our observations and emphasize the necessity of prolonged monitoring and reassessment in managing pediatric patients with VP [21].

Similarly, Benson et al. evaluated the spontaneous resolution of VP using transesophageal electrophysiological studies in a small cohort of very young infants, initially assessed at 2 months of age and followed up for an average of 12 months. They observed a rate of spontaneous resolution in the accessory pathways within the first year of life consistent with our data. However, Benson et al. did not evaluate the electrophysiologic properties of accessory pathways beyond early infancy into late childhood or adolescence. This gap highlights the need for further longitudinal studies to explore the persistence or resolution of these pathways over a more extended period [22].

Cain et al. employed a dichotomous statistical analysis to divide their pediatric WPW syndrome population into groups based on age at diagnosis and the presence of associated heart disease. While this method provides valuable insights into differences between these groups, particularly in risk stratification for SCD, it may not fully capture the dynamic nature of VP resolution over time [20]. The use of a statistical analysis that considers the left truncation of the data allowed us to depict for the first time a “real survival” curve of the anterograde conduction of the AP seen at ECG, from birth to 16 years of age. Left truncation correction is a statistical technique used in survival analysis that reduces the selection bias occurring when individuals who experienced the event prior to the beginning of the study (the age of diagnosis in this study) are not included in the study (left truncated data). This can occur when studying a time-dependent condition, such as the persistence of VP. This correction was used since most patients were recruited after the first days of life, which should be the right starting point for observing the persistence of VP, with VP being a congenital condition. Without accounting for left truncation data, the estimated “survival” probabilities would have been greatly overestimated, leading to incorrect conclusions about the persistence of VP over time.

More recently, Yammine et al. [23] retrospectively analyzed a single-center population of 67 patients with an accessory pathway who underwent invasive risk assessment with an electrophysiological study. They showed that 68.7% exhibited high-risk EP parameters. Moreover, there was no significant difference in AVRT inducibility between patients at high risk compared to those at low risk, and patients with significantly shorter AP ERP were more likely to be asymptomatic. On the basis of these data, they concluded that every pediatric patient with AP should be invasively evaluated.

However, some fundamental differences between their population and that of our study must be taken into account. First of all, the average age of our population is extremely low (even if in their population the age at diagnosis is not described), and the highest propensity for spontaneous disappearance of pre-excitation was in fact observed in our population at a much lower age than the average age analyzed by them. Secondly, their population was not analyzed in terms of evolution over time to assess any disappearance of pre-excitation; moreover, in our population, only 30% had undergone invasive assessment, so a direct comparison of the electrophysiological characteristics between the two populations is not feasible.

While catheter ablation can eliminate the risk of SCD, referring every child for an ablation could result in serious and potentially life-threatening complications [13,24]. Moreover, the assessment of AP conduction properties is more challenging and may potentially involve higher procedural risk [5,24]. The latest Consensus Statement on the management of this group of patients dates to 2012 but refers only to asymptomatic patients aged 8–21 years [13]. The most recent 2019 ESC guidelines on supraventricular tachycardia confirm previous 2012 indications [4]. Accordingly, in children over 8 years of age, non-invasive risk stratification with ECG monitoring and exercise testing is suggested and invasive assessment by means of transoesophageal and transvenous electrophysiological study is recommended for those deemed at higher risk. Recent findings further emphasize the limitations of non-invasive tests like the exercise stress test (EST) in accurately stratifying risk, demonstrating low sensitivity and negative predictive value in identifying high-risk accessory pathways, regardless of symptom presence. Their study advocates for the necessary inclusion of electrophysiological studies (EPSs) with the use of isoproterenol to improve diagnostic accuracy, aligning with the call for more definitive assessment techniques in high-risk pediatric cases [14,23,25].

In our population, excluding one patient who underwent ablation for refractory and hemodynamically impairing tachycardias, invasive evaluation or treatment was safely delayed after the age of 6, with an antiarrhythmic drug (mostly flecainide) being started when needed.

Our data significantly reinforce the hypothesis that conservative management should be the preferred choice in this group of patients, especially if diagnosed in the first years of life in asymptomatic and/or in patients with intermittent VP. In symptomatic younger children (below the age of 8 years), a pharmacological approach can be safely performed, postponing an invasive evaluation at an age when procedural risks are significantly reduced.

## 5. Limitations

The study’s limitations mainly lie in its retrospective nature, confounding factors, differential losses to follow-up, and incomplete data that could have altered the analysis results. The majority of VPs were detected following screening ECGs, thus limiting the applicability of our conclusions to children with tachycardia as the initial presentation. Moreover, the study could have been more precise with a higher number of patients recruited before the first year of life. However, the complexities of planning a prospective study are not negligible, as patients should be identified in the neonatal and infant period and followed for a long time frame. APs that lost anterograde conduction may maintain retrograde conduction properties, making them prone to maintaining AV orthodromic re-entry tachycardia. Lastly, it is important to note that it was not possible to exclude that some of our VP mechanisms may involve benign fasciculoventricular fibers that carry no risk of tachycardia or MAEs.

## 6. Conclusions

The management of VP in childhood remains challenging. Our study offers a unique insight into the natural history of VP from birth to adolescence. The probability of spontaneous resolution of VP during childhood is high, mostly in the first years of life. This suggests that conservative management should be preferred, especially in asymptomatic patients and those with intermittent VP. Our study highlights the rarity of major arrhythmic or life-threatening events in non-invasively treated patients, reinforcing this approach as a safe and viable option. However, the limitations of our retrospective study should be considered, and a larger prospective study would be needed to further validate these findings.

## Figures and Tables

**Table 1:** Characteristics of the patients (“d” stands for “days”, whereas the rest of the variables are expressed in years).

	Total (n = 153)	VP Persistence (n = 111)	VP Loss (n = 42)	*p*-Value
Female sex (n,%)	64 (41.8)	44 (39.6)	20 (47.6)	0.372
Age at diagnosis (years)	4.9 (75 d–8.4)	6 (1.3–9.2)	61 d (24 d–4.3)	<0.001
Follow-up time (days)	4.9 (1.7–8)	4.9 (1.6–7.2)	1954.5 (5.4–8.6)	0.550
CHD	8 (5.2)	6 (5.4)	2 (4.8)	0.121
Atrial septal defects (n,%)	3 (1.8)	1 (0.9)	2 (4.8)	0.122
Ventricular septal defects (n,%)	5 (3.3)	5 (4.5)	0 (0)	0.163
LV dyssynchrony (n,%)	8 (5.2)	6 (5.3)	2 (4.8)	0.897
Symptoms (n,%)	47 (30.7)	39 (35.1)	8 (19)	0.054
Palpitations	32 (68.1)	30 (76.8)	2 (25)	
Chest pain	1 (2.1)	1 (2.6)	0 (0)	
Dizziness	1 (2.1)	1 (2.6)	0 (0)	
Vomiting	3 (6.3)	1 (2.6)	2 (25)	
Poor oral intake	1 (2.1)	1 (2.6)	0 (0)	
Slow growth	1 (2.1)	0 (0)	1 (12.5)	
Heart failure	7 (14.9)	5 (12.8)	2 (25)	
SVT (n,%)	31 (20.3)	23 (20.7)	8 (19)	0.818

**Table 2:** Univariate and multivariate analyses.

Variants	Univariable Crude HR (95% CI)	Crude *p*-Value	Multivariable Adjusted HR (95% CI)	Adjusted *p*-Value
Female sex	1.22 (0.66–2–24)	0.522		
Intermittent VP	2.36 (0.91–6.12)	0.077	3.45 (1.32–9.05)	0.012
Non-invasive “low risk” characteristics	2.40 (0.79–7.26)	0.122		
Presence of symptoms	0.43 (0.20–0.93)	0.032	0.15 (0.03–0.68)	0.014
SVT	0.75 (0.35–1.64)	0.473		
Chronic drug therapy	0.82 (0.36–1.84)	0.619		
Left free wall AP	0.96 (0.43–2.15)	0.922		
Left and right free wall AP	0.93 (0.42–2.09)	0.866		
Free walls + epicardial AP	1.05 (0.51–2.17)	0.896		

**Table 3:** Other assessments (“d” stands for “days”, whereas the rest of the variables are expressed in years).

	Total (n = 153)	VP Persistence (n = 111)	VP Loss (n = 42)	*p*-Value
Non-invasive risk stratification (n, %)	112 (73.2)	93 (83.8)	19 (45.2)	<0.001
ECG Holter monitoring only (n, %)	61 (39.9)	48 (43.2)	13 (30.9)	
Exercise test only (n, %)	2 (1.3)	2 (1.8)	0 (0)	
Holter monitoring and ET (n, %)	49 (32)	43 (38.7)	6 (14.3)	
“Low risk” (n, %)	67 (43.8)	52 (46.8)	15 (35.7)	0.057
VP loss during exercise (n, %)	45 (29.4)	38 (34.2)	7 (16.7)	0.718
Intermittent VP (n, %)	22 (14.4)	14 (12.6)	8 (19)	0.005
AP localization				0.262
Right free wall (n, %)	33 (21.6)	26 (23.4)	7 (16.7)	
Right posteroseptal (n, %)	33 (21.6)	27 (24.3)	6 (14.3)	
Left posteroseptal (n, %)	6 (3.9)	6 (5.4)	0 (0)	
Anteroseptal (n, %)	19 (12.5)	11 (9.9)	8 (19.0)	
Left free wall (n, %)	21 (13.6)	17 (15.4)	4 (9.5)	
Epicardial (n, %)	12 (7.8)	9 (8.1)	3 (7.1)	
Indeterminate (n, %)	29 (18.9)	15 (13.5)	14 (33.4)	
Chronic drug strategy tested	27 (17.7)	20 (18.0)	7 (16.7)	0.845
None (n, %)	126 (82.3)	91 (82)	35 (83.4)	
1 strategy (n, %)	20 (13.1)	15 (13.5)	5 (11.8)	
2 strategies (n, %)	6 (3.9)	4 (3.6)	2 (4.8)	
3 strategies (n, %)	1 (0.7)	1 (0.9)	0 (0)	
Treatment starting age (years)	6.1 (27 d–11.1)	3.9 (22 d–11.1)	6.1 (1.1–11.1)	0.083
Invasive risk stratification (n, %)	46 (30)	41 (36.9)	5 (11.9)	0.002
Transoesophageal EPS (n, %)	9 (5.9)	9 (8.2)	0 (0)	0.056
Transvenous EPS (n, %)	42 (27.5)	37 (33.3)	5 (11.9)	0.080
Ablation (n, %)	30 (19.6)	30 (27)	0 (0)	
Ablation age (years)	12.7 (11.5–14.5)	12.7 (11.5–14.5)		
Major procedural complications	0 (0)	0 (0)		
Major arrhythmic events	0 (0)	0 (0)	0 (0)	

## Data Availability

Our data can be shared upon reasonable request made to our corresponding author (Dr. Rordorf).

## Supplementary Materials

The following supporting information can be downloaded at: https://www.mdpi.com/article/10.3390/jcm14072367/s1, Figure S1. Kaplan-Meier survival curves for VP persistence stratified by the presence of symptoms. Figure S2. Kaplan-Meier survival curves for VP persistence stratified by the presence of baseline risk assessment at non invasive risk stratification. Figure S3. Kaplan-Meier survival curves for VP persistence stratified by gender.

## Abbreviations

AF	Atrial fibrillation
AP	Accessory pathway
AV	Atrioventricular
CHD	Congenital heart disease
CI	Confidence interval
ECG	Electrocardiogram
EST	Exercise stress test
MAE	Major arrhythmic event
SCD	Sudden cardiac death
SD	Standard deviations
SPERRI	Shortest pre-excited RR interval
SVT	Supraventricular tachycardia
VP	Ventricular pre-excitation
WPW	Wolf–Parkinson–White
