# Supplementary material for: Spontaneous Resolution of Ventricular Pre-Excitation During Childhood: A Retrospective Study
Source: J Clin Med. 2025 Mar 29;14(7):2367. doi: 10.3390/jcm14072367 (PMC11989471; doi:10.3390/jcm14072367)

Supplementary material.

Figure S1. Kaplan-Meier survival curves for VP persistence stratified by the presence of symptoms

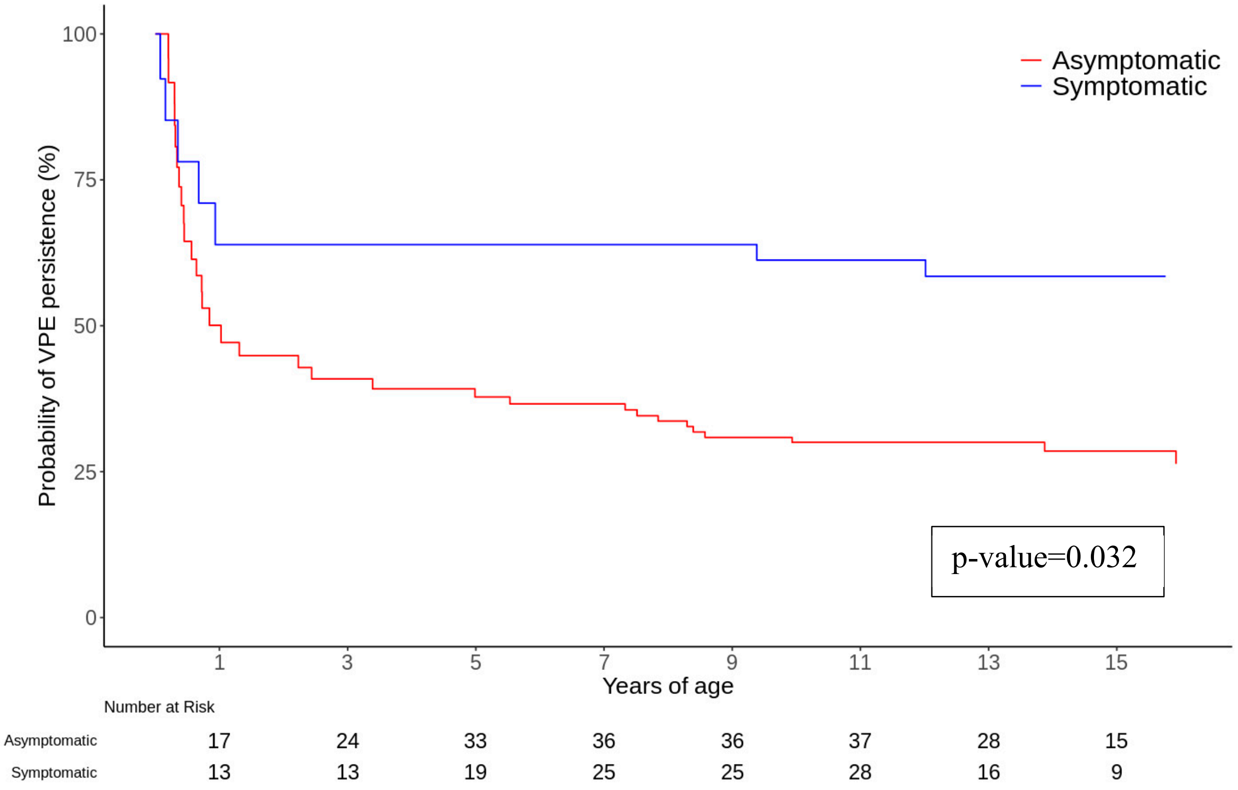

Figure S2. Kaplan-Meier survival curves for VP persistence stratified by the presence of baseline risk assessment at non invasive risk stratification.

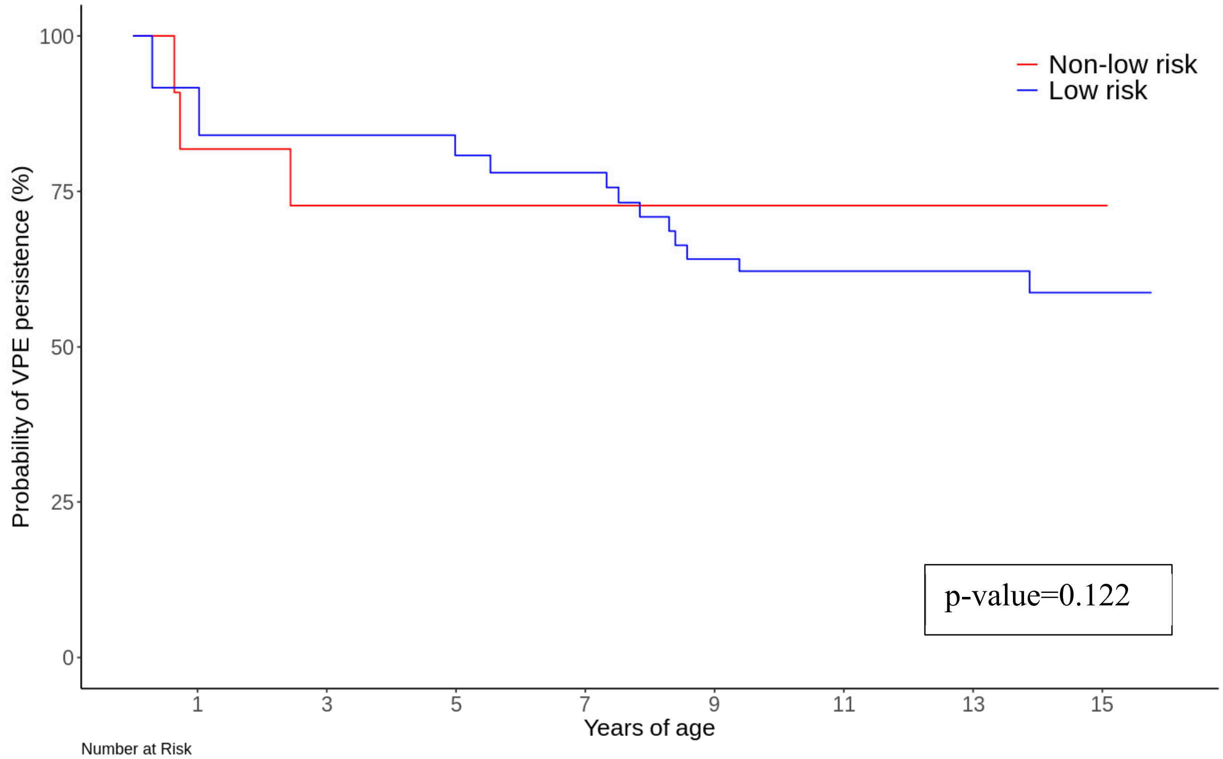

Number at Risk

	1	3	5	7	9	11	13	15
Non-low-risk	10	10	16	22	22	23	16	8
Low risk	12	23	29	33	33	35	22	14

Figure S3. Kaplan-Meier survival curves for VP persistence stratified by gender.

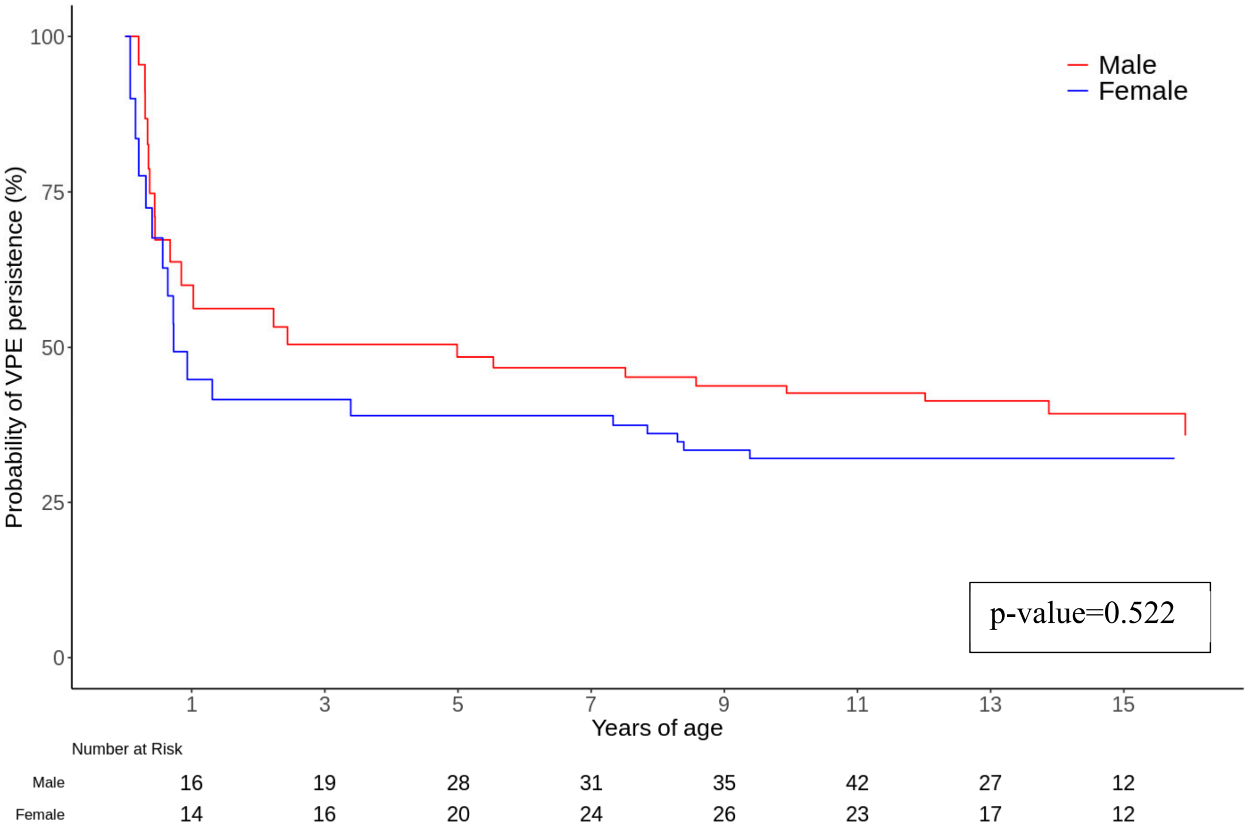

Supplement: Supplementary file 1 [file jcm-14-02367-s001.zip › jcm-3426382-supplementary.pdf]
